# Dysbiosis of the Urinary Bladder Microbiome in Cats with Chronic Kidney Disease

**DOI:** 10.1128/mSystems.00510-21

**Published:** 2021-07-27

**Authors:** Younjung Kim, Maura Carrai, Marcus H. Y. Leung, Jaime Chin, Jun Li, Patrick K. H. Lee, Julia A. Beatty, Dirk U. Pfeiffer, Vanessa R. Barrs

**Affiliations:** a Centre for Applied One Health Research and Policy Advice, Jockey Club College of Veterinary Medicine and Life Sciences, City University of Hong Kong, Hong Kong SAR; b Department of Infectious Diseases and Public Health, Jockey Club College of Veterinary Medicine and Life Sciences, City University of Hong Kong, Hong Kong SAR; c Centre for Companion Animal Health, Jockey Club College of Veterinary Medicine and Life Sciences, City University of Hong Kong, Hong Kong SAR; d Department of Veterinary Clinical Sciences, Jockey Club College of Veterinary Medicine and Life Sciences, City University of Hong Kong, Hong Kong SAR; e School of Energy and Environment, City University of Hong Kong, Hong Kong SAR; f Kowloon Cat Hospital, Hong Kong SAR; g Veterinary Epidemiology, Economics and Public Health Group, Department of Pathobiology and Population Sciences, The Royal Veterinary College, London, United Kingdom; Colorado State University

**Keywords:** *Escherichia coli*, microbiome, urinary tract infection, veterinary microbiology

## Abstract

Although feline urinary tract diseases cause high morbidity and mortality rates, and subclinical bacteriuria is not uncommon, the feline urinary microbiome has not been characterized. We conducted a case-control study to identify the feline urinary bladder microbiome and assess its association with chronic kidney disease (CKD), feline idiopathic cystitis (FIC), and positive urine cultures (PUCs). Of 108 feline urine samples subjected to 16S rRNA gene sequencing, 48 (44.4%) samples reached the 500-sequence rarefaction threshold and were selected for further analysis, suggesting that the feline bladder microbiome is typically sparse. Selected samples included 17 CKD, 9 FIC, 8 PUC cases and 14 controls. Among these, 19 phyla, 145 families, and 218 genera were identified. *Proteobacteria* were the most abundant, followed by *Firmicutes*. Notably, four major urotypes were identified, including two urotypes predominated by Escherichia*-Shigella* or *Enterococcus* and two others characterized by relatively high alpha diversity, Diverse 1 and Diverse 2. Urotype was associated with disease status (*P* value of 0.040), with the Escherichia*-Shigella*-predominant urotype being present in 53% of CKD cases and in all of the Escherichia coli PUC cases. Reflecting these patterns, the overall microbial composition of CKD cases was more similar to that of E. coli PUC cases than to that of controls (*P* value of <0.001). Finally, PUC cases had microbial compositions distinct from those of controls as well as CKD and FIC cases, with significantly lower Shannon diversity and Faith’s phylogenetic diversity values.

**IMPORTANCE** Despite the clinical importance of urinary diseases in cats, the presence of resident urine microbes has not been demonstrated in cats, and the role of these microbes as a community in urinary health remains unknown. Here, we have shown that cats with and without urinary tract disease harbor unique microbial communities in their urine. We found no evidence to suggest that the bladder microbiome is implicated in the pathogenesis of feline idiopathic cystitis, a disease similar to bladder pain syndrome/interstitial cystitis in humans. However, cats with chronic kidney disease had dysbiosis of their bladder microbiome, which was predominated by Escherichia*-Shigella* and had a community structure similar to that of cats with Escherichia coli cystitis. These findings suggest that chronic kidney disease alters the bladder environment to favor Escherichia*-Shigella* colonization, potentially increasing the risk of overt clinical infection.

## INTRODUCTION

The application of high-throughput sequencing methodologies has provided convincing evidence that the urine of healthy humans contains unique bacterial communities, collectively termed the urinary microbiome (1–3). It was subsequently demonstrated that most of the bacterial taxa detected by 16S rRNA gene sequencing in the female urinary microbiome also grew under enhanced culture conditions, confirming that these bacteria indeed resided in the lower urinary tract (4). Distinct urinary microbial communities, i.e., urotypes, have also been identified in humans, based on similarities in urinary microbiome composition (4, 5) or the presence of predominant bacterial taxa (6, 7).

The association of the urinary microbiome with various urinary and reproductive tract diseases in humans, including painful bladder syndrome/interstitial cystitis (PBS/IC) (6, 8–12), urinary incontinence (4), bladder cancer (13), prostatitis (14), and chronic kidney disease (CKD) (5), has been investigated. Some of these studies suggest that similar to host-associated microbial communities in other sites (e.g., the gut and skin microbiomes), urinary microbes play important roles in both the maintenance of health and the development of disease through complex interactions with the host.

Given the proven existence of the urinary microbiome in humans and dogs (15, 16), it seems reasonable to hypothesize that a feline urinary microbiome also exists and may interact differently with the host depending on the health of the urinary system. Certainly, subclinical bacteriuria is not uncommon in cats (17), and urinary tract diseases, notably CKD and feline idiopathic cystitis (FIC), are common causes of morbidity and mortality in cats. However, previous investigations have failed to characterize a urinary microbiome in cats. In one study, feline urine samples and a negative-extraction control had similar abundance patterns of bacterial sequence reads, indicating likely bacterial contamination of the urine samples (18). In another investigation, no feline urine samples yielded positive sequence results, likely due to methodological limitations (19). The methodological and technical constraints in these studies likely reflect that the bacterial load of most urine is extremely low and show the difficulty in sequencing such low-abundance microbial communities.

CKD is a common disorder in geriatric cats, characterized by an irreversible and progressive loss of kidney function and affecting at least 30 to 40% of cats aged over 10 years (20). Also, secondary urinary tract infections (UTIs) are a frequent complication of feline CKD, especially in females (17). FIC is an idiopathic disorder of cats with lower urinary tract signs (LUTS), which has similarities to PBS/IC in women (21). For both CKD and FIC, the exclusion of UTIs by routine urine culture is a fundamental component of diagnostic evaluation, with the assumption that resident bacteria play no role in health maintenance or disease development in cats with negative culture results. However, in cats with CKD, the urine’s physical and biochemical properties are changed, thereby presenting an altered environment for bacteria in the lower urinary tract. Due to impaired urine-concentrating ability, cats with CKD typically have dilute urine (urine specific gravity [USG] of <1.035) and decreased urine pH (≤6.0) (22). In contrast, no definitive pathognomonic features have been identified in the urine of cats with FIC, a disorder linked to an imbalance between the sympathetic nervous system and the hypothalamic-pituitary-adrenal axis (21). Considering the associations between host-associated microbial communities and the nervous system (e.g., the microbiota-gut-brain axis) (23), cats with FIC might harbor distinct bacterial communities associated with the development of LUTS via nervous system stimulation.

In this study, we hypothesized that cats harbor a urinary microbiome and that urotypes, diversity and composition are distinguishable between cats with and without CKD, FIC, or positive urine cultures (PUCs). To test these hypotheses, we characterized the feline bladder microbiome and investigated its association with CKD, FIC, and PUC based on 16S rRNA gene hypervariable V4 region sequencing. A case-control study was conducted among cats attending first-opinion veterinary clinics in Hong Kong to (i) characterize the diversity and composition of the feline bladder microbiome among cats with and without CKD, FID, or PUC; (ii) identify different urotypes; and (iii) determine whether urotypes, diversity, and composition were associated with CKD, FIC, or PUC.

## RESULTS

### Basic characteristics of study samples.

Between April and September 2020, 108 residual urine samples were collected from the bladder via cystocentesis (suprapubic aspiration) from four first-opinion veterinary clinics in Hong Kong. After reviewing the rarefaction curve, 48 samples with more than 500 sequences were included in the analysis (referred to here as “study samples”) (see Fig. S1 in the supplemental material). As expected, all samples from PUC cases met the rarefaction threshold and were included. However, other disease status, demographics, urine pH, and specific gravity were not associated with meeting the rarefaction threshold (Table S1). The study samples originated from 14 controls and 17 CKD, 9 FIC, and 8 PUC cases (Table 1). Among CKD cases, 10, 5, and 2 were diagnosed as having stage 2, stage 3, and stage 4 CKD, respectively. The distributions of demographic variables, urine pHs, and USGs between study groups are summarized in Table 1. Among PUC cases, both Escherichia coli and Enterococcus faecalis were isolated from one cat, and only E. coli or E. faecalis was isolated from the remaining cats (5 and 2 cats, respectively). At the time of enrollment, while all 6 PUC cases with E. coli isolates had LUTS reported by the owners, LUTS were not reported in one PUC case with E. faecalis isolated.

**TABLE 1:** Baseline characteristics of study groups

Variable	Value for group								*P* value^*a*^
	Control		CKD		FIC		PUC		
	No.	%	No.	%	No.	%	No.	%	
Sex									
Female	4	28.6	7	41.2	3	33.3	8	100.0	0.020
Male	10	71.4	10	58.8	6	66.7	0	0.0	

Age (yrs)									
<6	4	28.6	1	5.9	5	55.6	2	25.0	0.020
≥6 and <12	7	50.0	3	17.6	3	33.3	2	25.0	
≥12	3	21.4	13	76.5	1	11.1	4	50.0	

Breed									
DSH^*c*^	7	50.0	13	76.5	5	55.6	3	37.5	0.307
Others	7	50.0	4	23.5	4	44.4	5	62.5	

Neutered									
Yes	12	85.7	16	94.1	9	100.0	7	87.5	0.674
No	2	14.3	1	5.9	0	0.0	1	12.5	

Urine pH									
<6.0	2	14.3	9	52.9	1	11.1	2	25.0	0.230
≥6.0 and <7.0	9	64.3	7	41.2	6	66.7	6	75.0	
≥7.0	3	21.4	1	5.9	2	22.2	0	0.0	

USG									
<1.035	2	14.3	17	100	1	11.1	7	87.5	NA^*b*^
≥1.035	12	85.7	0	0.0	8	88.9	1	12.5	


Total	14	100	17	100	9	100	8	100	

a Fisher’s exact test *P* values were FDR adjusted to account for multiple testing.

b NA, not applicable. Statistical testing was not performed as USG was one of the criteria used to define CKD cases.

c DSH, domestic short hair.

10.1128/mSystems.00510-21.1 FIG S1 Rarefaction curve based on Shannon diversity. Different line colors represent different samples. The gray vertical line represents the rarefaction threshold to select study samples (500 sequences). Download FIG S1, PDF file, 0.3 MB. Copyright © 2021 Kim et al. 2021 Kim et al. https://creativecommons.org/licenses/by/4.0/ This content is distributed under the terms of the Creative Commons Attribution 4.0 International license.

10.1128/mSystems.00510-21.4 TABLE S1 Comparison of variables between urine samples that met the rarefaction threshold (included) and those that did not (excluded). Download Table S1, DOCX file, 0.02 MB. Copyright © 2021 Kim et al. 2021 Kim et al. https://creativecommons.org/licenses/by/4.0/ This content is distributed under the terms of the Creative Commons Attribution 4.0 International license.

### Taxonomic classification of 16S rRNA gene sequences.

After denoising and decontamination, 161,868 sequences were dereplicated to 690 unique amplicon sequence variants (ASVs) among study samples. Among 13 extraction-negative controls, the maximum number of sequence reads was 61, with no sequences detected in 6 extraction-negative controls (Fig. S2). A total of 8 genera were identified across all extraction-negative controls. The number of genera per extraction-negative control ranged from 0 to 3, with a median of 1. *Lactococcus*, *Lachnoclostridium*, and the Ruminococcus gauvreauii group were identified as potentially contaminating bacteria by decontam, based on their detection frequencies in all 108 samples and extraction-negative controls (24). Therefore, sequences assigned to these taxa were removed from the sequence data, along with nonbacterial 16S rRNA gene sequences (i.e., mitochondria and chloroplasts). The number of sequences per sample ranged between 502 and 20,481, with a median of 2,082.

10.1128/mSystems.00510-21.2 FIG S2 Distribution of urine (*n* = 108) and extraction-negative control (*n* = 13) samples by the number of sequences after quality control. Download FIG S2, PNG file, 0.4 MB. Copyright © 2021 Kim et al. 2021 Kim et al. https://creativecommons.org/licenses/by/4.0/ This content is distributed under the terms of the Creative Commons Attribution 4.0 International license.

**FIG 1:**
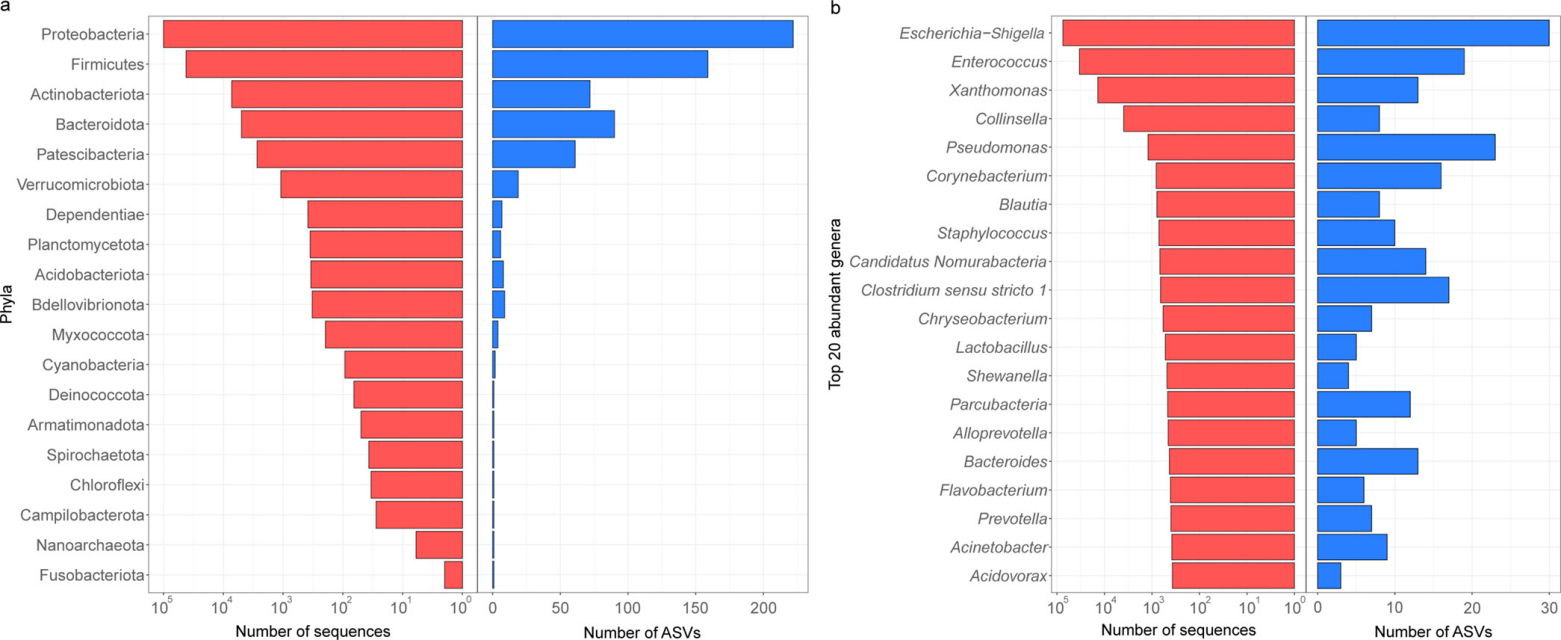
Taxonomic classification of ASVs. Phylum-level (a) and genus-level (b) taxonomic classifications are shown. In each panel, the right bar chart (in blue) shows the distribution of ASVs assigned to different taxa, and the left bar chart (in red) shows the number of sequences dereplicated to these ASVs.

**FIG 2:**
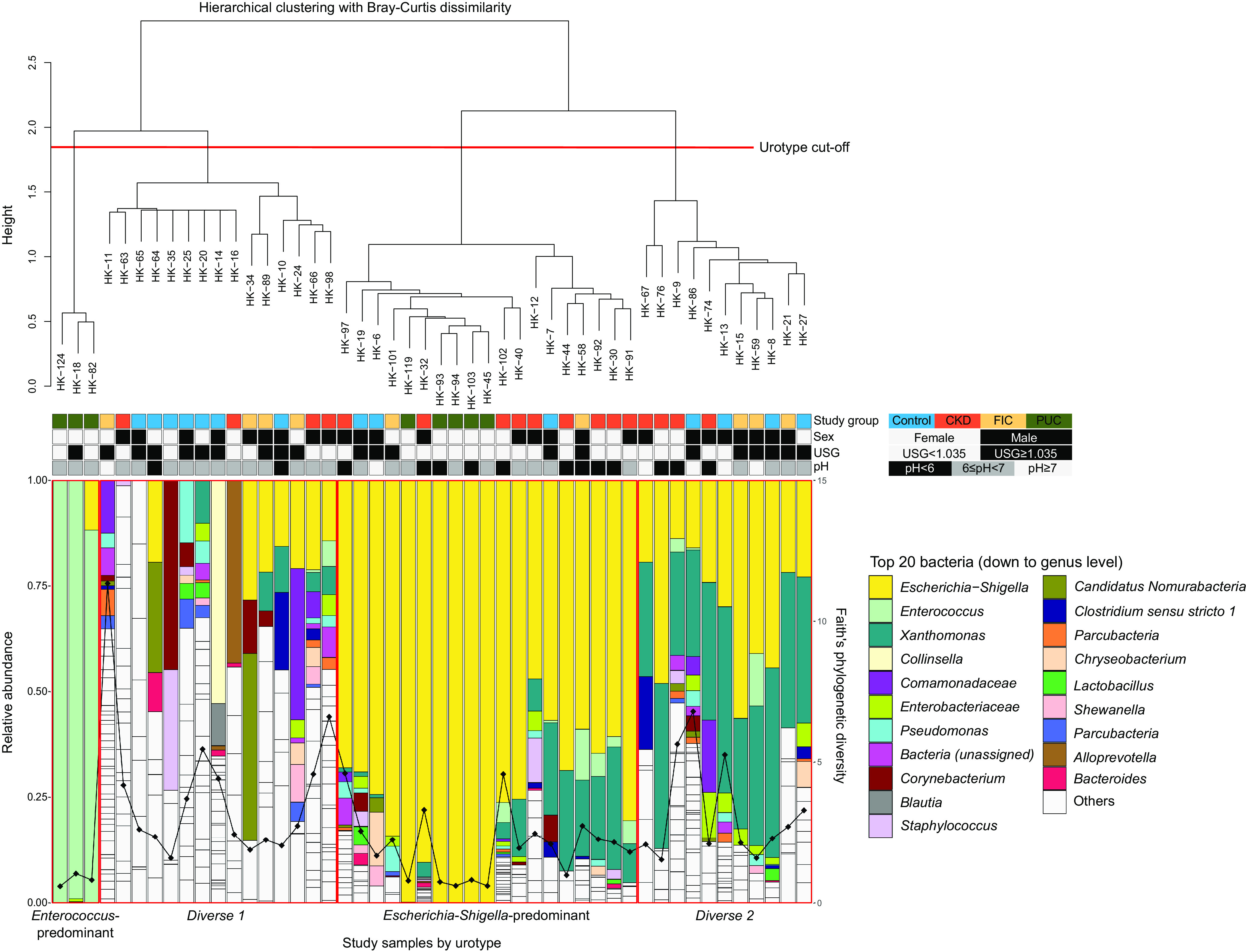
Bacterial composition of study samples and urotypes. Study samples are ordered by urotype defined based on the results of hierarchical clustering with Bray-Curtis dissimilarity, as shown by the dendrogram. In the bar chart, vertical bars represent the relative abundances of bacterial taxa identified within individual samples, with nonwhite colors representing the 20 most abundant bacterial taxa. Points on the bar chart represent individual samples’ Faith phylogenetic diversity. Horizontal bars above the bar chart show the study group, sex, USG, and urine pH of individual samples with different colors.

The ASVs were assigned to 19 phyla, 145 families, and 218 genera using the naive Bayes classifier (25) (via q2‐feature‐classifier) against the SILVA 138 99% 515F and 806R region reference sequences (26). On average, 10 different genera were identified per cat, with an interquartile range of between 1 and 38. All 8 PUC cases had fewer than 6 genera, i.e., the 1st quartile, along with 1 CKD case and 1 FIC case. At the phylum level, *Proteobacteria* were the most abundant, followed by *Firmicutes* (Fig. 1a). At the genus level, Escherichia*-Shigella* and *Enterococcus* comprised most of the *Proteobacteria* sequences and *Firmicutes* sequences, respectively (Fig. 1b). Escherichia*-Shigella* and *Xanthomonas* were the most prevalent genera among study samples (Fig. S3). Escherichia*-Shigella* and/or *Enterococcus* sequences predominated in PUC samples, consistent with their culture findings. However, while 60.2% of Escherichia*-Shigella* sequences originated from around three-quarters of non-PUC cats, 94.9% of *Enterococcus* sequences originated from only three PUC cases with E. faecalis isolates, and the remaining *Enterococcus* sequences were detected in only around a quarter of non-PUC cats (Fig. 2).

10.1128/mSystems.00510-21.3 FIG S3 Proportions of study samples with individual genera. A sample is determined as having a given genus if the sample had at least one sequence dereplicated to an ASV assigned to that genus. For a given genus, the *y* axis shows the proportion of samples determined as having that genus. Download FIG S3, PDF file, 0.03 MB. Copyright © 2021 Kim et al. 2021 Kim et al. https://creativecommons.org/licenses/by/4.0/ This content is distributed under the terms of the Creative Commons Attribution 4.0 International license.

### Alpha and beta diversity values between study groups.

**FIG 3:**
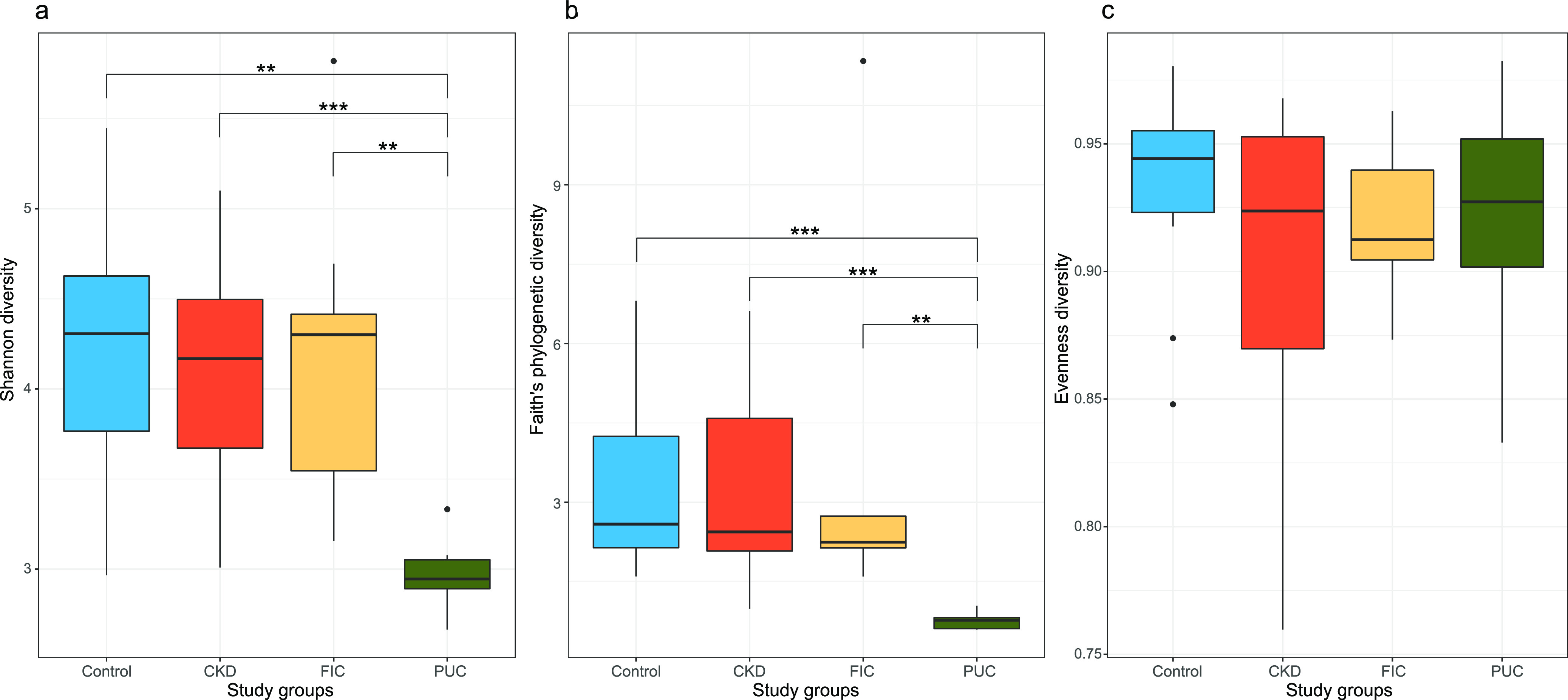
Comparison of alpha diversity metrics between study groups. (a) Shannon diversity; (b) Faith’s phylogenetic diversity; (c) evenness. For each pair of study groups, the Kruskal-Wallis rank sum test is used to assess the statistical significance of alpha diversity differences. FDR-adjusted Kruskal-Wallis rank sum test *P* values are shown (***, *P* value of <0.001; **, *P* values of ≥0.001 and <0.01). *P* values of ≥0.05 are not shown.

Microbial diversity within samples (alpha diversity) was assessed by Shannon diversity, Faith phylogenetic diversity, and evenness. Briefly, evenness quantified how “equally” individual ASVs were present in numbers within samples. Shannon diversity extended evenness by quantifying how “much” and equally individual ASVs were present within individual samples. Faith phylogenetic diversity was represented by the sum of the branch lengths of a phylogenetic tree connecting all ASVs. There was no statistical evidence that CKD and FIC cases had alpha diversity values different from those of the controls, as assessed by all alpha diversity metrics (Fig. 3). However, PUC cases had significantly lower alpha diversity than controls and other study groups, as determined by Shannon diversity and Faith phylogenetic diversity (Fig. 3a and b), while there was no association with evenness (Fig. 3c).

**FIG 4:**
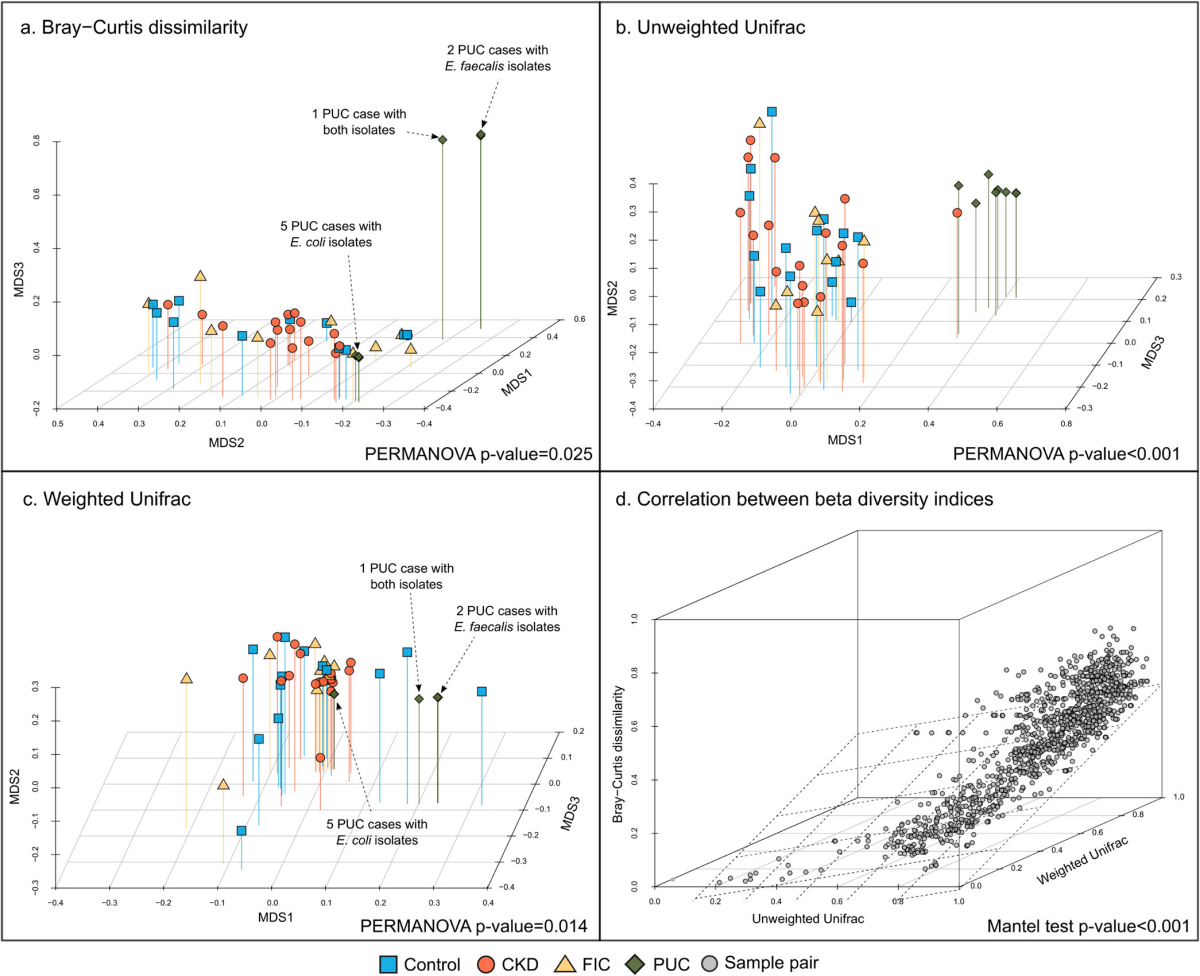
Comparison of beta diversity metrics between study groups. (a to c) Samples are ordinated in reduced space using multidimensional scaling (MDS) based on Bray-Curtis dissimilarity (a), unweighted UniFrac (b), and weighted UniFrac (c). Permutational multivariate analysis of variance (PERMANOVA) is used to assess the statistical significance of beta diversity between study groups. (d) The Mantel test is used to assess the correlation between these beta diversity metrics.

Bray-Curtis dissimilarity, unweighted UniFrac, and weighted UniFrac values were computed for each pair of samples to assess their differences in microbiome composition (beta diversity). While Bray-Curtis dissimilarity accounted for ASV abundance, UniFrac was computed based on a phylogenetic tree’s branch lengths shared between samples, with (weighted) and without (unweighted) accounting for ASV abundance. Beta diversity analysis revealed that the bladder microbiome composition of PUC cases was different from those of the controls and CKD and FIC cases, as visualized by multidimensional scaling (MDS) ordinations (Fig. 4a to c) and assessed by permutational multivariate analysis of variance (PERMANOVA) (Table 2). However, there was no evidence of a compositional difference in the bladder microbiomes between controls and CKD and FIC cases (Table 2). There was strong evidence that Bray-Curtis dissimilarity, unweighted UniFrac distance, and weighted UniFrac distance were positively correlated (*P* value of <0.001 for each pair of beta diversity metrics) (Fig. 4d).

**TABLE 2:** Statistical significance of the differences in beta diversity between study groups

Comparison groups	FDR-adjusted PERMANOVA *P* value^*a*^		
	Bray-Curtis	Unweighted UniFrac	Weighted UniFrac
Control vs CKD	0.172	0.732	0.366
Control vs FIC	0.647	0.942	0.940
Control vs PUC	**0.047**	**<0.001**	0.090
CKD vs FIC	0.899	0.899	0.636
CKD vs PUC	0.096	**<0.001**	**0.009**
FIC vs PUC	0.202	**<0.001**	**0.002**

Overall	**0.025**	**<0.001**	**0.014**

a *P* values of <0.05 are highlighted in boldface type.

### Urotypes of the feline urinary bladder microbiome.

**FIG 5:**
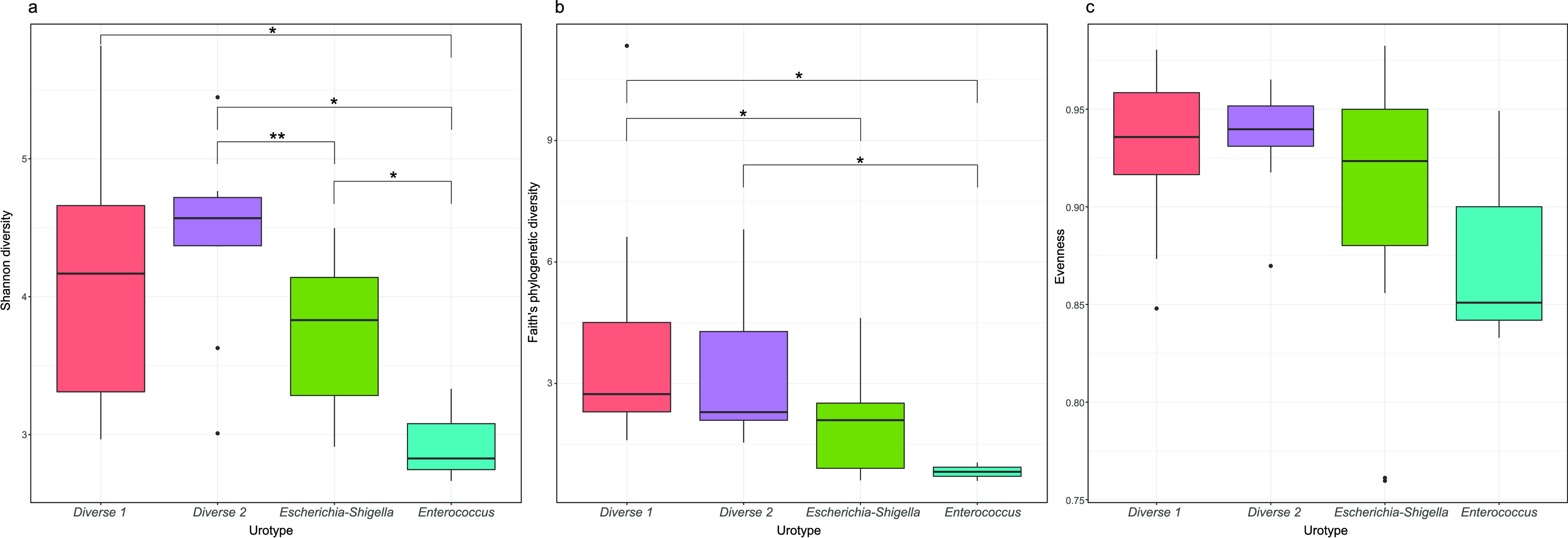
Comparison of alpha diversity metrics between urotypes. (a) Shannon diversity; (b) Faith’s phylogenetic diversity; (c) evenness. FDR-adjusted Kruskal-Wallis rank sum test *P* values are shown (**, *P* values of ≥0.001 and <0.01; *, *P* values of ≥0.01 and <0.05). *P* values of ≥0.05 are not shown. The Diverse 1 urotype was associated with higher Faith’s phylogenetic diversity than both the Escherichia*-Shigella*-predominant (mean difference, 1.83 [95% confidence interval {CI}, 0.35 to 3.31]; *P* value of 0.034) and *Enterococcus-*predominant (mean difference, 3.06 [95% CI, 1.65 to 4.48]; *P* value of 0.034) urotypes. The Diverse 2 urotype also showed a similar trend in alpha diversity, associated with higher Shannon diversity than those of both the Escherichia*-Shigella-*predominant (mean difference, 0.71 [95% CI, 0.23 to 1.18]; *P* value of 0.009) and *Enterococcus-*predominant (mean difference, 1.48 [95% CI, 0.81 to 2.15]; *P* value of 0.043) urotypes.

**FIG 6:**
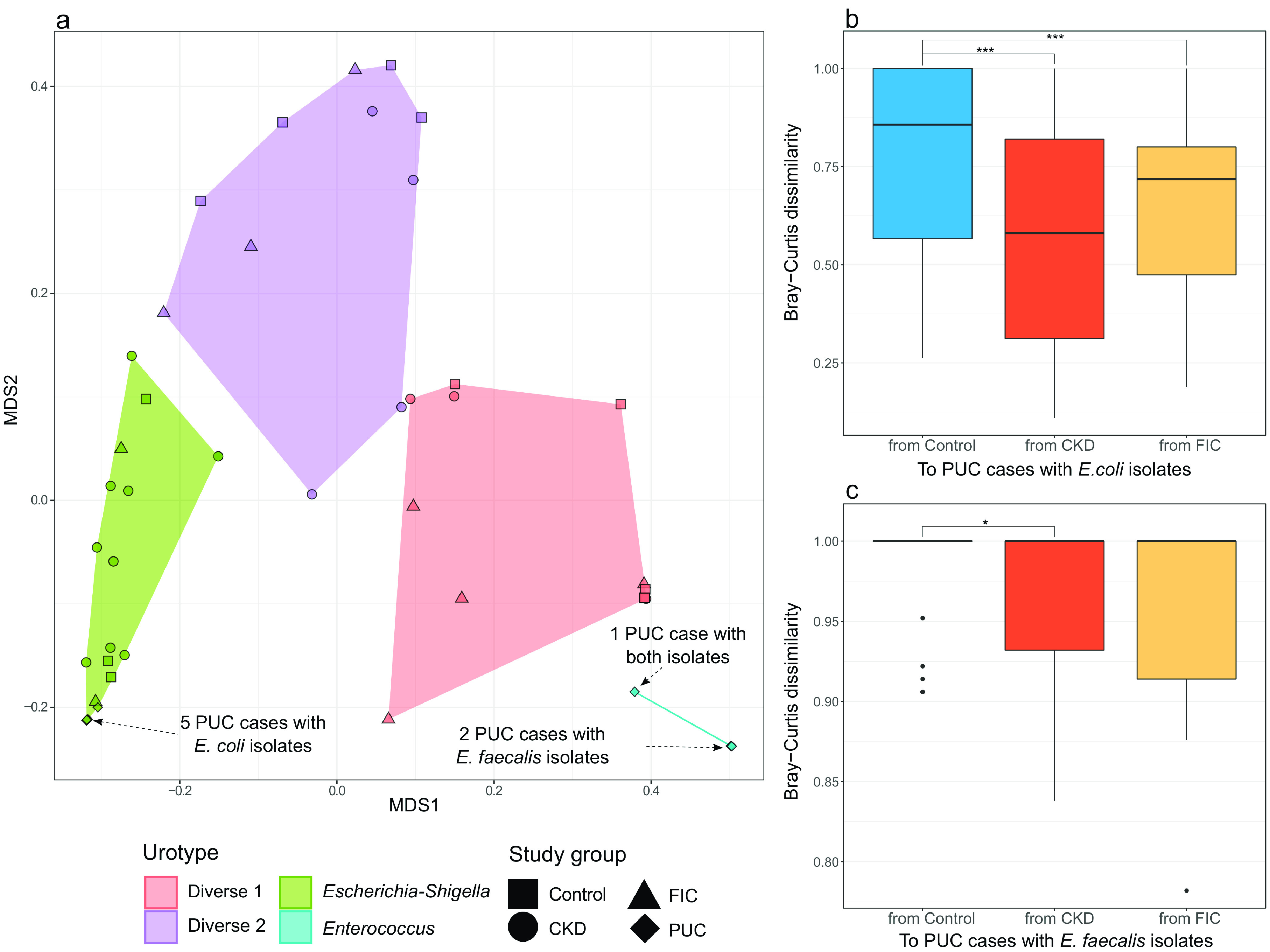
Compositional similarity of the urinary bladder microbiome by urotype and study group. (a) Study samples are ordinated in reduced space using multidimensional scaling (MDS) based on Bray-Curtis dissimilarity. Points represent individual samples, with different shapes representing different study groups. Samples of different urotypes are highlighted in different colors. (b and c) The box plots on the right show the distribution of the Bray-Curtis dissimilarity indices from controls and CKD and FIC cases to PUC cases with Escherichia coli isolates (b) and to those with Enterococcus faecalis isolates (c). The Bray-Curtis dissimilarity index ranges from 0 to 1, with higher values indicating more dissimilar microbial compositions between two given samples. FDR-adjusted Kruskal-Wallis rank sum test *P* values are shown (***, *P* value of <0.001; *, *P* values of ≥0.01 and <0.05). *P* values of ≥0.05 are not shown.

Based on compositional microbiome similarities, study samples were classified into one of the following four urotypes: Diverse 1 (n = 15), Diverse 2 (*n* = 11), Escherichia*-Shigella* predominant (*n* = 19), and *Enterococcus* predominant (*n* = 3) (Fig. 2). Both the Diverse 1 and Diverse 2 urotypes consisted of similar numbers of taxa. However, while 12 different bacterial taxa represented the most abundant taxa for individual Diverse 1 urotype cats, only 2 taxa, Escherichia*-Shigella* and *Xanthomonas*, did so for individual Diverse 2 urotype cats (Table 3), resulting in the Diverse 2 urotype being nested within the same clade as the Escherichia*-Shigella*-predominant urotype (Fig. 2). The Diverse 1 and Diverse 2 urotypes were characterized by higher alpha diversity than the Escherichia*-Shigella*-predominant and *Enterococcus*-predominant urotypes (Fig. 5). Within the Escherichia*-Shigella*-predominant urotype, PUC cases with E. coli isolates were highly clustered (Fig. 6a). Escherichia*-Shigella* sequences comprised 46.9% to 100% (median, 75.4%) of sequence reads in the Escherichia*-Shigella*-predominant urotype cats, and *Enterococcus* sequences comprised between 88.3% and 99.8% (median, 98.9%) among the *Enterococcus*-predominant urotype cats. These patterns were in stark contrast to those of cats with the Diverse 1 and Diverse 2 urotypes, whose most abundant bacterial taxa comprised on average 22.2% and 36.8% of the sequences, respectively (Table 3).

**TABLE 3:** Characteristics of urotypes by the number of taxa and the most abundant taxa identified in individual samples^*a*^

Urotype	No. of cats	No. of taxa (range)	Most abundant taxon		
			Relative abundance (%) (range)	No.	Taxon name(s)
Diverse 1	15	11 (5–46)	22.2 (11.0–52.7)	12	*Actinomyces*, *Comamonadaceae*, *Collinsella*, *Alloprevotella*, *Atopostipes*, *Psychrobacter*, “*Candidatus* Nomurabacteria,” *Corynebacterium*, *Massilia*, *Aquaspirillum*, Escherichia*-Shigella*, *Anaerococcus*
Diverse 2	11	9 (7–32)	36.8 (24.5–56.3)	2	Escherichia*-Shigella*, *Xanthomonas*
Escherichia *-Shigella*	19	9 (1–27)	75.4 (46.9–100)	1	Escherichia *-Shigella*
*Enterococcus*	3	3 (2–4)	98.9 (88.3–99.8)	1	*Enterococcus*

a Taxonomic classification was made down to the genus level. Numbers in parentheses represent the ranges between the minimum and maximum values.

The distribution of the studied cats among urotypes was associated with the cats’ disease status. First, the classification of PUC cases based on the sequence data was consistent with their culture results. All 5 PUC cases with only E. coli isolates were classified into the Escherichia*-Shigella*-predominant urotype. One PUC case with both E. coli and E. faecalis isolates was predominated by Escherichia*-Shigella* and *Enterococcus* sequences and classified into the *Enterococcus*-predominant urotype due to a higher abundance of *Enterococcus* than Escherichia*-Shigella*, together with 2 PUC cases with only E. faecalis isolates. Interestingly, more than half of the CKD cases were classified into the Escherichia*-Shigella*-predominant urotype, and nearly 80% of the controls and FIC cases were classified into the Diverse 1 or Diverse 2 urotype (*P* value of 0.040) (Table 4). This finding was in line with the observation that PUC cases with E. coli isolates tended to share a more similar microbiome composition with CKD cases than with controls (Fig. 6b and c).

**TABLE 4:** Characteristics of urotypes

Characteristic	Value [no. (%)]					*P* value^*a*^
	Total	Diverse 1	Diverse 2	Escherichia *-Shigella*	*Enterococcus*	
Study group						
Control	14	7 (50.0)	4 (28.6)	3 (21.4)	0	0.040
CKD	17	4 (23.5)	4 (23.5)	9 (52.9)	0	
FIC	9	4 (44.4)	3 (33.3)	2 (22.2)	0	
PUC	8	0	0	5 (62.5)	3 (37.5)	

Sex						
Female	22	6 (27.3)	3 (13.6)	10 (45.5)	3 (13.6)	0.251
Male	26	9 (34.6)	8 (30.8)	9 (34.6)	0	

Age (yrs)						
<6	12	3 (25.0)	4 (33.3)	4 (33.3)	1 (8.3)	0.470
≥6, <12	15	5 (33.3)	5 (33.3)	4 (26.7)	1 (6.7)	
≥12	21	7 (33.3)	2 (9.5)	11 (52.4)	1 (4.8)	

Urine pH						
<6.0	14	2 (14.3)	3 (21.4)	9 (64.3)	0	0.103
≥6.0, <7.0	28	12 (42.9)	4 (14.3)	9 (32.1)	3 (10.7)	
≥7.0	6	1 (16.7)	4 (66.7)	1 (16.7)	0	

USG						
<1.035	27	6 (22.2)	5 (18.5)	14 (51.9)	2 (7.4)	0.261
≥1.035	21	9 (42.9)	6 (28.6)	5 (23.8)	1 (4.8)	

a Fisher’s exact test *P* values were FDR adjusted to account for multiple testing.

## DISCUSSION

Here, we characterized the feline urinary bladder microbiome using culture-independent methods. Four different urotypes were identified based on the bladder microbiome composition of cats with and without CKD, FIC, or PUC. Notably, urotype classification was associated with disease status. Controls or FIC cases tended to harbor urotypes comprised of diverse bacterial taxa. In contrast, Escherichia*-Shigella* predominated the bladder microbiome in over half of the CKD cases and all PUC cases with E. coli isolates. As expected, PUC cases showed significantly different bladder bacterial diversity and composition from those of the other study groups.

### Diverse and distinct urotypes in control cats.

The finding of diverse urotypes in the majority of controls, especially the Diverse 1 urotype, where no predominant taxa were identified, is in marked contrast to the human urinary microbiome, where predominance of particular taxa has been commonly observed. For example, *Lactobacillus* most frequently predominates in the urinary microbiome of women (4, 7, 27–29). *Lactobacillus* has also been frequently detected at relatively high abundances in the urinary microbiome of some men (1, 28, 30), although this was not the case in males undergoing genitourinary surgery procedures (31) or with sexually transmitted infections (1). However, despite the use of similar methodologies, these genera were detected in only a few feline urine samples, regardless of disease status.

Considering that host and environmental influences play a significant role in determining the microbiome composition (32), it is not surprising that the studied cats harbored bladder bacterial communities distinct from those of humans, as observed in comparative microbiome studies of the gut (33), skin (34), and vagina (35). One reason for the difference in *Lactobacillus* abundances would be that *Lactobacillus* generally exists at low abundances in the vagina of nonhuman mammals (36–38). While the feline genital microbiome has not yet been characterized using culture-independent methods, *Lactobacillus* was isolated in only 3% of feline vaginal samples in a culture-dependent study (39). In contrast, *Lactobacillus* is the most predominant bacterial taxon in the human vaginal microbiome, observed in both culture-dependent (40) and culture-independent (41) studies.

Another factor likely to have influenced the observed differences from the human urinary microbiome is the urine collection technique. We used cystocentesis, which presents the least risk of bacterial contamination from external environments, whereas almost all human studies used catheterized (3, 27) or voided (5, 28, 42, 43) urine samples, which are prone to bacterial contamination from the genital tract. In the only human urinary microbiome study to use cystocentesis, the relative abundance of *Lactobacillus* in women was significantly higher in catheterized urine samples than in urine samples collected by cystocentesis (27.0% and 6.9%, respectively), although it was still the most abundant bacterium in the latter sample type (3).

Our findings also suggest that the feline bladder microbiome is different from the canine bladder microbiome. Notably, while Pseudomonas was detected at relatively low abundances in around half of the control cats, Pseudomonas predominated the urine of both healthy female and male dogs (15). Considering that Pseudomonas also predominated the genital microbiome in most of these dogs (15), this genus was likely a true member of the bladder microbiome with close connections to the genital microbiome, like *Lactobacillus* in women. However, in a culture-dependent study of cats, Pseudomonas was not isolated from samples collected from the genital tract (39), consistent with its less frequent and abundant detection in our study. In contrast to humans and dogs, Escherichia*-Shigella* was detected in most of the cats in this study, although its relative abundance tended to be much lower in the diverse urotypes, especially the Diverse 1 urotype, than in the Escherichia*-Shigella*-predominant urotype. Considering that E. coli is one of the most common causes of UTI in cats (44), such frequent detection of Escherichia*-Shigella* suggests that E. coli is commonly present, albeit in a relatively low abundance, in the feline urinary tract in health and that cats become at risk of UTI development when the urinary tract environment becomes suitable for its proliferation.

Finally, the above-described differences in the feline bladder microbiome compared to those of humans and dogs were also likely influenced by the unique nutritional requirements of felids. As obligate carnivores, cats require a diet that primarily consists of protein and fat, unlike the omnivorous diets of humans and dogs (45). Considering that urine excretes by-products from nutrient metabolism, including those of amino acids, the urine of cats likely presents different environmental conditions to urine microbes, compared with the urine of humans and dogs.

### Predominance of Escherichia*-Shigella* in the urine of cats with chronic kidney disease.

While the bladder microbiome of control cats tended to be represented by diverse taxa, over half of the CKD cases and all five PUC cases with only E. coli isolates were classified into the urotype predominated by Escherichia*-Shigella*. Escherichia*-Shigella* predominance among CKD cases was in contrast to previous human studies where only a minority of the participants with and without urinary or genital tract diseases had E. coli predominance (28), Escherichia*-Shigella* or Escherichia (7, 27), or *Enterobacteriaceae* (4, 5). In particular, the Escherichia*-Shigella-*predominant urotype was observed in only 3.9% of old adults with CKD, while diverse urotypes were most frequently observed in these CKD patients, followed by *Corynebacterium*-predominant and Staphylococcus-predominant urotypes (5).

Interestingly, three of five PUC cases with Escherichia*-Shigella* predominance also had CKD as the underlying condition but were classified as PUC cases due to E. coli isolation. The presence of CKD in the remaining two PUC cases could not be confirmed as serum biochemistry was not available. However, these cats also exhibited reduced urine-concentrating ability, which is a hallmark of, and most commonly caused by, CKD. Together with the frequent observation of Escherichia*-Shigella* predominance in CKD cases, these findings suggest that the bladder environment of cats suffering from CKD is likely more favorable to Escherichia*-Shigella* than other bacteria prone to clinical urinary tract infection. In particular, these findings may explain why CKD is one of the most common systemic comorbidities in cats with UTIs (46), from the perspective of the urinary microbiome. The pathogenesis of CKD directly influences the urine’s physical and biochemical properties. As CKD progresses, the kidneys’ impaired urine-concentrating ability decreases urine concentration toward isosthenuria and increases the urinary excretion of hydrogen ions in response to metabolic acidosis, as previously observed in CKD cats (22). Such changes could alter the bladder environment, favoring certain bacteria and potentially leading to their overgrowth and, eventually, dysbiosis in the bladder microbiota. Indeed, E. coli was found to have an increased ability to bind to host cells when the pH was low (47). Also, from *in vitro* culture of three different E. coli isolates from dogs, Thornton et al. (48) showed that the level of E. coli growth was higher in more dilute urine and neutral-to-acidic urine. Additionally, urine with E. coli isolates exhibited the lowest pHs among samples collected from pediatric human patients (49). These findings are in line with our observations that around three-quarters of cats with the Escherichia*-Shigella* urotype, including all PUC cases with E. coli isolates, had dilute urine (USG of <1.035), and all but one with this urotype had acidic urine.

Our findings suggest that the bladder microbiome diversity and composition, not just the presence of causative bacteria, play a role in UTI development in cats. While over half the CKD cases were classified into the same urotype as PUC cases with E. coli isolates, CKD cases still tended to have higher alpha diversity than PUC cases. Considering that they were also culture negative, our findings suggest that despite Escherichia*-Shigella* predominance, E. coli growth in the bladder of these cats was not sufficient to result in overwhelming dysbiosis with positive culture results. These observations then raise the question of what conditions could have triggered the overgrowth, not just the predominance, of E. coli sufficient to cause PUCs in some CKD cases. Older female cats are at a higher risk of developing UTIs among cats with CKD (17). Also, Price et al. (7) showed that the urinary microbiome was more likely to be predominated by Escherichia in older women. These observations are consistent with ours in that the three PUC cases that had CKD as the underlying condition were all females aged over 14 years. Considering also that old cats generally have a weak immune system, our findings suggest that biological factors associated with sex and age may play a role in predisposing the bladder microbiome in CKD cases to overgrowth and potentially overt clinical infection. Whether E. coli-associated UTIs in cats with CKD are mostly invasions (e.g., from the gastrointestinal or genital tract) or blooms of resident members of the urinary microbiome is unknown and warrants further investigation.

### No identified association of urine microbes with feline idiopathic cystitis.

FIC is often compared to PBS/IC in women due to their clinicopathological similarities (21). Human urinary microbiome studies on PBS/IC have conflicting results, with some investigators finding no evidence for a microbial contribution to the disorder (6, 10, 12) and others suggesting a link (8, 9, 11). In our study, most FIC cases had a diverse urotype, along with most control cats. These findings indicate that the bladder environment of cats with FIC is not susceptible to the growth of certain bacterial taxa and suggest that bacteria are unlikely to play a major role in FIC pathogenesis.

### Detection of other microbes.

In this study, other common causative bacteria of feline UTIs, including *Enterococcus*, Pseudomonas, Staphylococcus, and Streptococcus (46, 50), were also frequently detected in non-PUC cats, including control cats. However, except for *Enterococcus* in PUC cases with E. faecalis isolates, these genera were less prevalent and, when present, less abundant than the levels observed with Escherichia*-Shigella*. Moreover, no species belonging to these genera were isolated by routine culture, suggesting that the bladder environment was not suitable for their overgrowth in the studied cats. Interestingly, PUC cases with only E. faecalis isolates did not have CKD, with normal or close-to-normal urine concentration, whereas one PUC case with both E. faecalis and E. coli isolates had severely dilute urine. This may suggest that E. coli and E. faecalis may favor different but not mutually exclusive environmental conditions.

*Xanthomonas* was the second most predominant genus in this study. Belonging to the family *Xanthomonadaceae* of the phylum *Proteobacteria*, it is known to cause diseases in different types of plants (51). Other than plants, its isolation has been reported only in insects (52), birds, and bats (51), which were assumed to transmit *Xanthomonas* to plants. In the only two studies of the canine bladder microbiome, genera belonging to the family *Xanthomonadaceae* were commonly detected in the urine of healthy dogs. Melgarejo et al. (16) detected *Xanthomonas*, along with *Stenotrophomonas* and *Bradyrhizobium*. Burton et al. (15) also detected *Xanthomonadaceae*, but when we reanalyzed their sequence data using the bioinformatics protocols used in this study, most of them were assigned to *Bradyrhizobium* and *Stenotrophomonas*, and none were assigned to *Xanthomonas*. Importantly, *Xanthomonas*, together with *Bradyrhizobium* and *Stenotrophomonas*, has been frequently detected in extraction-negative controls, with molecular-biology-grade water, PCR reagents, and DNA extraction kits as possible sources (53). Therefore, one possible explanation for *Xanthomonas* detection could be that it was a contaminant introduced during DNA extraction. We did not remove these genera because they were not detected in any of the extraction-negative controls but were detected only in study samples across almost all extraction batches. However, while contaminating bacteria are more likely to be detected in extraction-negative controls than in true samples due to the absence of competing DNA (24), this may not be the case for samples with low biomass, like urine. Therefore, although our study took measures to avoid potential contamination, including filter sterilization and the inclusion of extraction-negative controls, we still could not exclude the possibility that they were contaminants.

### Strengths and limitations.

Several factors likely contributed to the successful identification of the feline bladder microbiome in this study. Considering that the bacterial load of most urine is typically very low, samples were transported on ice and frozen within a relatively short period of time after collection (≤3 h) to minimize bacterial DNA degradation. This was particularly important because the study was designed to use residual diagnostic urine samples, and obtaining a large volume of urine to compensate for low biomass was thus not feasible. Also, to maximize bacterial DNA yields, samples were treated with additional lysis buffers proven effective in human urinary microbiome studies (42). Finally, an extraction-negative control was added to each extraction batch to facilitate the identification of potentially contaminating bacteria, which could easily overwhelm low-abundance microbial communities.

There were also several limitations in our study. First, we did not investigate the genital microbiome of the studied cats because this study was designed to use residual urine collected for diagnostic purposes. The proximity of the lower urinary and genital tracts suggests that the genital tract is likely to serve as a source of urinary bacteria. Previous human studies showed that while the bladder microbiome was dissimilar to that of the urethra, periurethra, and voided urine (54), it shared highly similar functional capabilities with the vaginal microbiome (55). Therefore, characterization of the feline genital microbiome would have allowed a more comprehensive interpretation of our findings, as discussed above. Second, we could not control for age and sex when assessing the association of the bladder microbiome with the cats’ disease status. Age is a well-known risk factor for CKD in cats (56). Reflecting this, CKD cases were relatively older than controls, making it difficult to exclude the possibility that age was a confounder. Notably, age-related factors, such as a relatively weak immune system in old cats, could have contributed to classifying the majority of CKD cases into the Escherichia*-Shigella*-predominant urotype, with or without the effect of CKD-related factors, including urine pH and USG. Also, sex has been associated with FIC, with males at a higher risk (57). Considering also that female and male urinary microbiome compositions are known to differ in humans (13, 30), controlling for sex, together with age, would have provided more robust study results. Finally, it was likely that this study lacked statistical power after the removal of samples with sequencing depths that were too low. When the alpha and beta diversity values of study groups were compared directly without considering their urotypes, there was no statistical evidence that CKD and FIC cases had bladder microbial communities different from those of the controls. However, when the study groups were compared by urotypes, the majority of the CKD cases were classified into the Escherichia*-Shigella*-predominant urotype, and most controls were classified into the Diverse 1 and Diverse 2 urotypes. These findings suggest that a lack of statistical evidence between study groups could have occurred due to the small sample size rather than no actual association. In particular, both CKD and FIC have a broad disease spectrum. While we included only cats that were not on dietary intervention for CKD to control for its impact, considering that the CKD cases in our study were experiencing different stages of CKD, the influence of CKD on the bladder environment could have varied depending on disease severity. Cats with FIC could also have multiple different pathogenic mechanisms of disease since the diagnosis is made by exclusion. For these reasons, the microbial diversity and composition of CKD and FIC cases could still be heterogeneous, hampering the detection of associations with a small sample size. In this study, a large number of samples were removed due to insufficient sequencing depth, indicating that feline urine typically has low biomass. Therefore, future studies could be improved by using a larger urine volume and focusing on a specific age or sex stratum when a large sample size is not attainable.

### Conclusion.

The findings here suggest that cat urine contains microbial communities distinct from those of humans and dogs. The identified urotypes were associated with the studied cats’ disease status. While the urine of controls and FIC cases likely consisted of diverse bacterial taxa, Escherichia*-Shigella* tended to predominate the urine of CKD cases along with PUC cases with E. coli isolates, and *Enterococcus* predominated the urine of PUC cases with E. faecalis isolates. Therefore, our findings show a possible underlying transition in the bladder microbiome before overt clinical infection develops and highlight the need for incorporating the view on feline urine as a living microbial ecosystem into the current diagnostic approach to feline urinary tract diseases.

## MATERIALS AND METHODS

### Study design.

This study was designed as a case-control study targeting cats attending first-opinion veterinary clinics. The study population consisted of cats attending four veterinary hospitals for first-opinion veterinary consultation. The study used residual urine samples collected for diagnostic purposes with owner consent and was exempted from animal ethics approval by the Animal Research Ethics Sub-committee, City University of Hong Kong. Cats were recruited for the study only if urinalysis and urine culture were performed on a sample collected via ultrasound-guided cystocentesis. Cats were excluded if there was a history of systemic antimicrobial administration or urinary catheter placement in the last 3 months or if insufficient residual urine was available for 16S rRNA gene sequencing. Case and control cats were defined as follows. CKD cases had (i) urine specific gravity (USG) of <1.035, (ii) a serum creatinine concentration of >140 μmol/ml, (iii) a negative urine culture result, and (iv) no dietary intervention for CKD. CKD cases were staged according to International Renal Interest Society guidelines (58). FIC cases were cases presenting with one or more of the LUTS, including pollakiuria, stranguria, periuria, dysuria, and hematuria, where diagnostic investigation, including physical examination, urinalysis, urine culture, and abdominal ultrasonography, failed to identify a specific cause (21). Cases with a positive urine culture result (i.e., the growth of >1,000 CFU/ml) were defined as PUC cases. Cats were defined as control cats if (i) they presented for reasons other than CKD or LUTS, (ii) they had no history of LUTS in the last 3 months, and (iii) the diagnostic test results showed no evidence of CKD, feline lower urinary tract diseases, and PUC.

Residual urine for 16S rRNA gene sequencing was stored at 4°C in the clinic, transported to the City University of Hong Kong on ice, and frozen at −80°C until DNA extraction within 3 h of collection.

### DNA extraction and 16S rRNA gene sequencing.

DNA extraction, PCR amplification, and 16S rRNA gene sequencing were performed using previously described protocols in human urinary microbiome studies (42). Briefly, DNA extraction was performed in a pre-PCR laminar flow hood. After thawing on ice, 1 ml of urine was centrifuged at 17,000 × *g* for 10 min at 4°C. Next, 900 μl of the supernatant was discarded. The remaining pellet was resuspended with 200 μl of lysis buffer 1, consisting of 20 mM Tris-HCl, 2 mM EDTA, 1.20% Triton X-100, and 20 μg/μl lysozyme, and 30 μl of lysis buffer 2, consisting of 20 mM Tris-HCl, 2 mM EDTA, and 5 U/μl mutanolysin, to enhance the lysis of Gram-positive as well as Gram-negative bacteria (59). Both lysis buffers 1 and 2 were reconstituted with ultrapure water (Invitrogen, CA, USA), filter sterilized before use to minimize the risk of bacterial contamination, and added to urine samples as well as extraction-negative controls. Next, genomic DNA was extracted with the DNeasy blood and tissue kit (Qiagen, Germany) according to the manufacturer’s protocol. Extracted DNA was stored at −80°C. For every 9 urine samples, lysis buffers 1 and 2 were added to an empty 1.5-ml collection tube and mock extracted to identify potentially contaminating bacterial taxa (i.e., extraction-negative control).

The DNA samples were sent to Loyola Genomics Facilities, Loyola University Chicago (Maywood, IL, USA), for 16S rRNA gene sequencing. Hypervariable region 4 (V4) of the 16S rRNA gene was amplified with a two-step PCR protocol. In the first step, the V4 region was amplified with Illumina MiSeq modified universal primers 515F and 806R, including both extraction-negative controls and PCR-negative controls. In the second step, the reaction mixtures were diluted 1:50 and amplified for 10 more cycles using primers with the adapter sequences for Illumina MiSeq sequencing and an 8-nucleotide sample index. The reaction mixtures were purified with Agencourt AMPure XP-PCR magnetic beads (Beckman Coulter, Pasadena, CA, USA) after each amplification and normalized to a standard concentration based on the DNA concentration measured by the Qubit fluorometer system (Thermo-Fisher, Waltham, MA, USA). Finally, the reaction mixtures were pooled and sequenced in the 2- by 250-bp sequencing reagent cartridge, according to the manufacturer’s instructions (Illumina, San Diego, CA, USA).

### Characterization of the feline urinary bladder microbiome.

The bioinformatics analysis of 16S rRNA gene sequence data was performed with QIIME 2 2020.11 (60). Raw paired-end sequences were denoised, trimmed at 200 bases, merged, and dereplicated into ASVs with the DADA2 plug-in (61) (q2‐dada2). Taxonomy was assigned to ASVs using the naive Bayes classifier (25) (via q2‐feature‐classifier) against the SILVA 138 99% 515F and 806R region reference sequences (26). ASVs were aligned with mafft (66) (via q2‐alignment), and the resulting alignment was used to construct a phylogenetic tree with fasttree2 (62) (via q2‐phylogeny). Since urine contains low microbial biomass in the absence of active infection, its sequence data are often subject to contamination. Thus, we identified potentially contaminating bacterial taxa based on their detection frequency in samples and extraction-negative controls using the prevalence-based method of the decontam package (24) in R.4.0.2 (63) and removed ASVs assigned to these bacterial taxa as well as mitochondria and chloroplasts from the sequence data.

Different ASV counts, i.e., the total number of sequences dereplicated into ASVs, are likely to reflect different sequencing depths rather than real biological variations among samples. Therefore, the sequencing depth of individual samples should be sufficient to represent their microbial communities. To review this, we assessed how Shannon diversity, one of the alpha diversity metrics, changed over different ASV counts on a rarefaction curve. We identified an ASV count from which Shannon diversity began to saturate in most samples and chose samples whose ASV count was above this threshold as “study samples,” assuming that the sequencing depth was sufficient in these samples to represent their urinary bladder microbiome. Samples with ASV counts below this threshold were excluded from this study.

With study samples, we first characterized the diversity and composition of the feline bladder microbiome among study samples. We computed evenness, Shannon diversity, and Faith phylogenetic diversity to assess ASV diversity within individual samples (alpha diversity). Next, we computed Bray-Curtis dissimilarity, unweighted UniFrac, and weighted UniFrac for each pair of samples to assess their compositional microbiome dissimilarity (beta diversity). For each beta diversity metric, we performed multidimensional scaling (MDS) using the cmdscale function in R.4.0.2 (63) and, based on its results, ordinated samples in reduced space to visualize their compositional microbiome dissimilarities. The above-described alpha and beta diversity metrics were computed using q2‐diversity of QIIME 2 2020.11 (60) after samples were subsampled without replacement to the lowest ASV count among study samples.

Second, we identified different urotypes by classifying samples into different groups based on their microbiome composition. More specifically, we quantified compositional microbiome dissimilarity for each pair of samples with Bray-Curtis dissimilarity. We then constructed a dendrogram where samples were grouped based on Bray-Curtis dissimilarity using Ward’s hierarchical clustering method (64). We defined urotypes by cutting the dendrogram at the starting point of the longest branch. Each of these urotypes was named after the taxon whose median relative abundance was >50% among samples of that urotype or, if there was no taxon satisfying this criterion, as “diverse.” We then assessed the distribution of study groups and other variables between urotypes with Fisher’s exact test.

Finally, we investigated the association of the diversity and composition of the feline bladder microbiome with CKD, FIC, and PUC based on the identified urotypes as well as the above-described alpha and beta diversity metrics. We compared each of the studied alpha diversity metrics between study groups and assessed its statistical significance with the Kruskal-Wallis rank sum test. Also, for each of the studied beta diversity metrics, we performed PERMANOVA with 99,999 permutations to assess its statistical significance between study groups. We also assessed if Bray-Curtis dissimilarities from PUC samples with E. coli overgrowth and those with *Enterococcus* overgrowth differed depending on the group of samples compared using the Kruskal-Wallis rank sum test. False discovery rate (FDR)-adjusted *P* values are reported in this study to account for multiple-hypothesis testing.

### Data availability.

The sequence data that support the findings of this study are available in the NCBI Sequence Read Archive (SRA) under BioProject accession number PRJNA719002 (65).
