# Supplementary figures and images for: Dysbiosis of the Urinary Bladder Microbiome in Cats with Chronic Kidney Disease
Source: mSystems. 2021 Jul 27;6(4):e00510-21. doi: 10.1128/mSystems.00510-21 (PMC8407359; doi:10.1128/mSystems.00510-21)

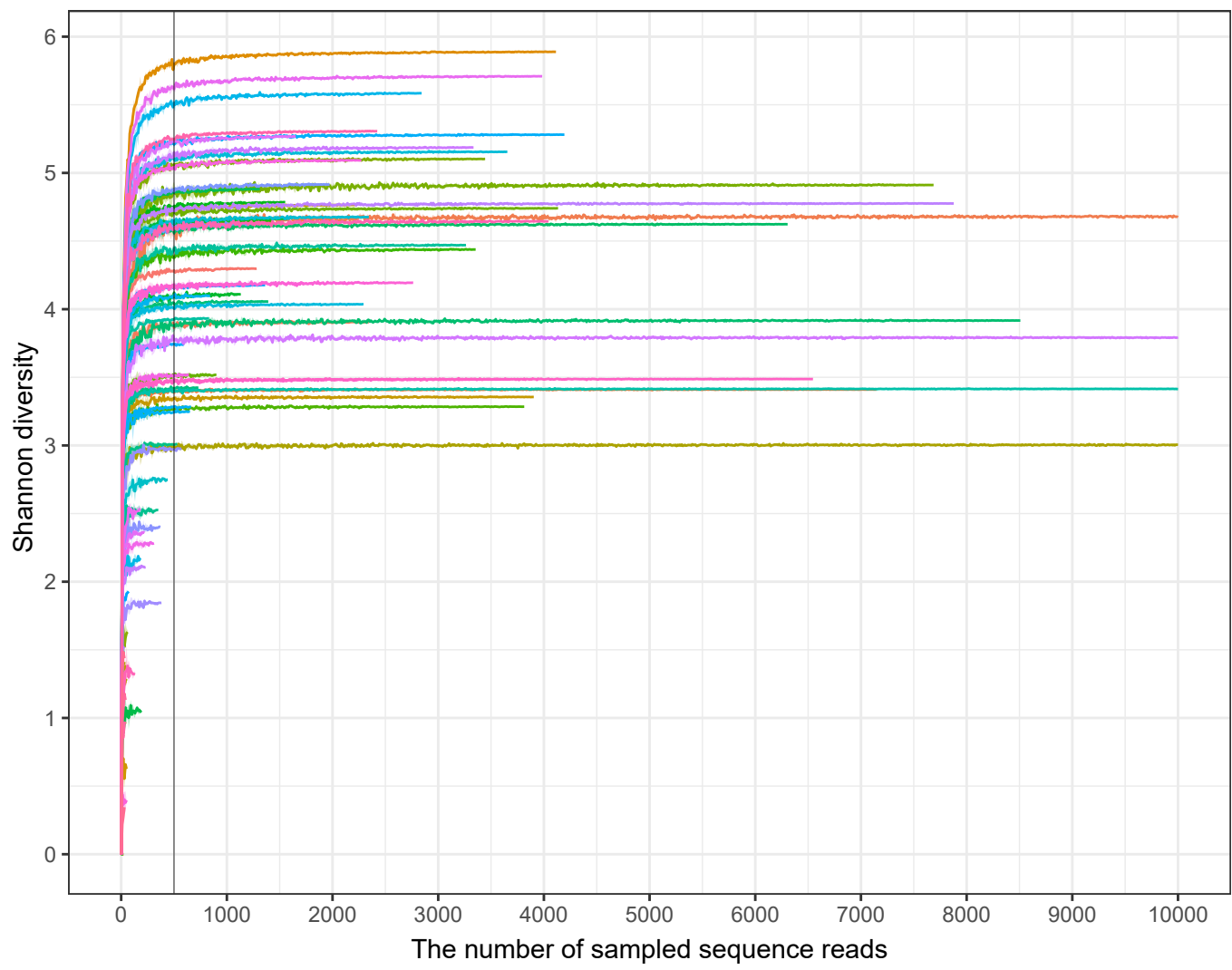

Supplement: FIG S1 [file msystems.00510-21-sf001.pdf]

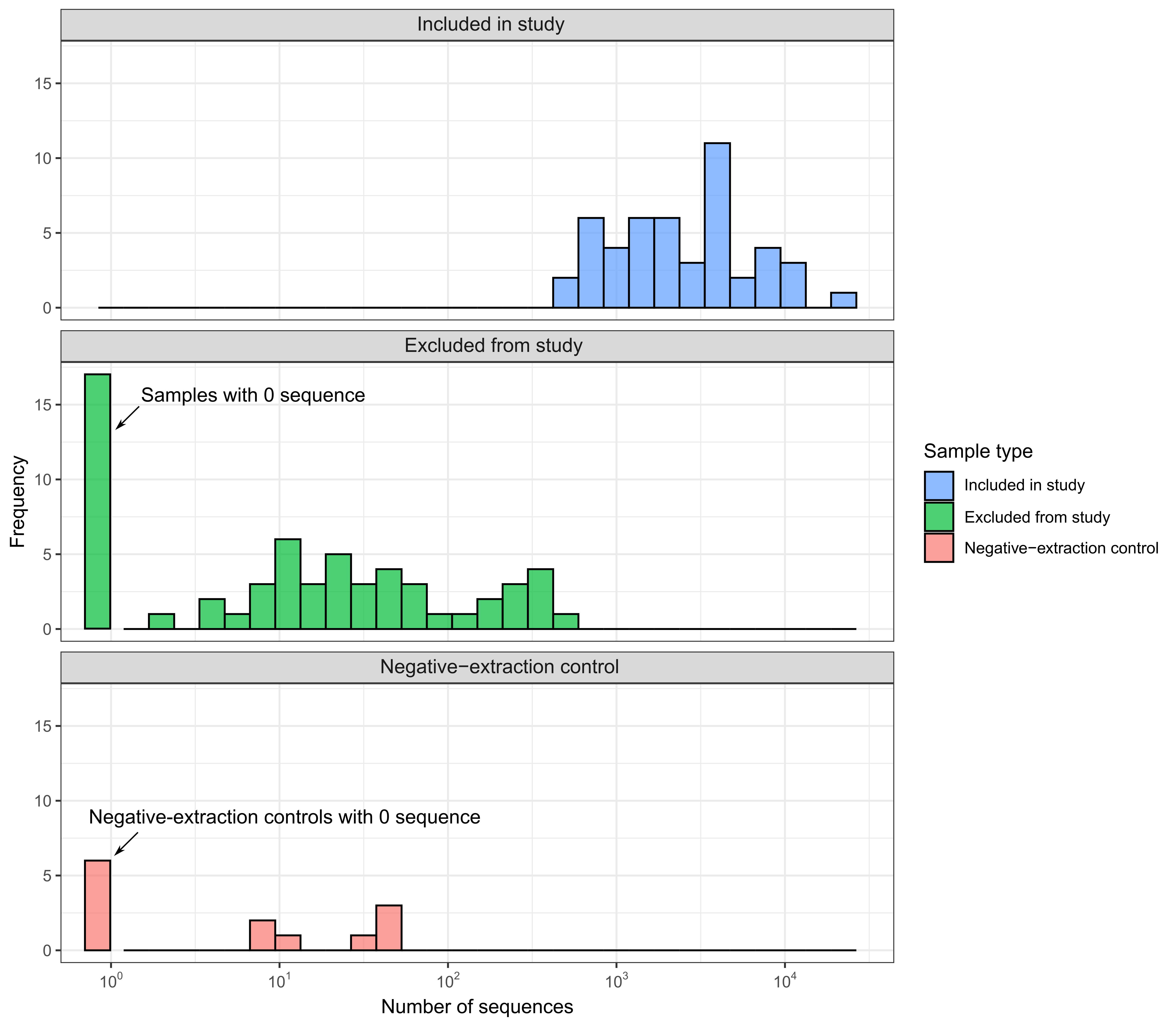

Supplement: FIG S2 [file msystems.00510-21-sf002.png]

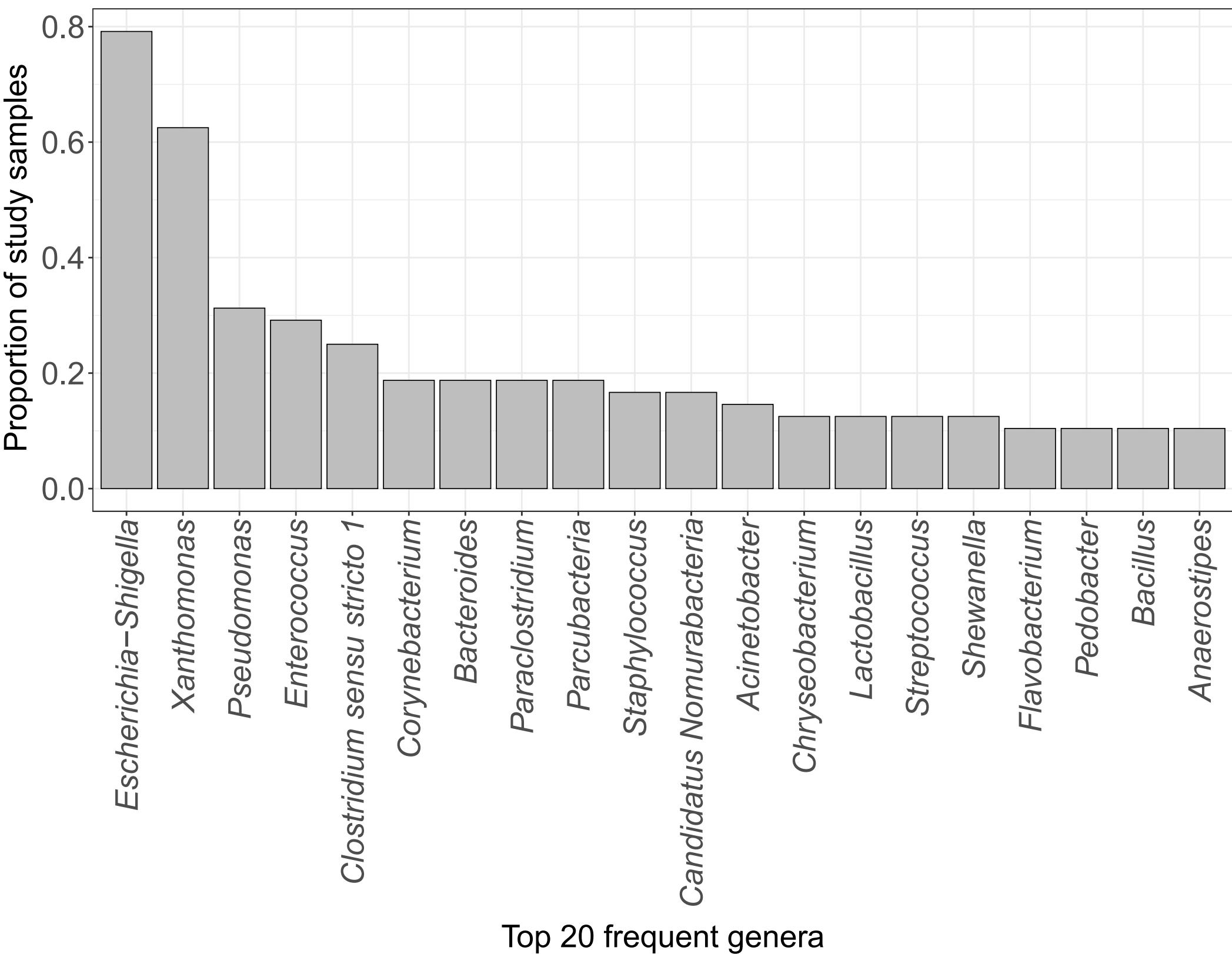

Supplement: FIG S3 [file msystems.00510-21-sf003.pdf]
