# Supplementary material for: Dysbiosis of the Urinary Bladder Microbiome in Cats with Chronic Kidney Disease
Source: mSystems. 2021 Jul 27;6(4):e00510-21. doi: 10.1128/mSystems.00510-21 (PMC8407359; doi:10.1128/mSystems.00510-21)
Supplement: TABLE S1 [file msystems.00510-21-st001.docx]

**Table S1** Comparison of variables between urine samples that met the rarefaction-threshold (included) and those that did not (excluded)

| **Variables** | **Study samples [no. (%)]** | | | | ***P* value^a^** |
| --- | --- | --- | --- | --- | --- |
|  | **Excluded** | | **Included** | |  |
| **Study group** |  |  |  |  |  |
| Control | 25 | (64.1) | 14 | (35.9) | 0.015 |
| CKD | 29 | (63.0) | 17 | (37.0) |  |
| FIC | 6 | (40.0) | 9 | (60.0) |  |
| PUC | 0 | (0.0) | 8 | (100.0) |  |
| **Sex** |  |  |  |  |  |
| Female | 29 | (56.9) | 22 | (43.1) | 0.848 |
| Male | 31 | (54.4) | 26 | (45.6) |  |
| **Age (in years)** |  |  |  |  |  |
| <6 | 8 | (40.0) | 12 | (60.0) | 0.438 |
| ≥6 and <12 | 26 | (63.4) | 15 | (36.6) |  |
| ≥12 | 26 | (55.3) | 21 | (44.7) |  |
| **Breed** |  |  |  |  |  |
| DSH | 28 | (50.0) | 28 | (50.0) | 0.438 |
| Others | 32 | (61.5) | 20 | (38.5) |  |
| **Neutered** |  |  |  |  |  |
| Yes | 59 | (57.3) | 44 | (42.7) | 0.438 |
| No | 1 | (20.0) | 4 | (80.0) |  |
| **Urine pH** |  |  |  |  |  |
| <6.0 | 14 | (50.0) | 14 | (50.0) | 0.658 |
| ≥6.0 and <7.0 | 34 | (54.8) | 28 | (45.2) |  |
| ≥7.0 | 12 | (66.7) | 6 | (33.3) |  |
| **USG** |  |  |  |  |  |
| <1.035 | 30 | (52.6) | 27 | (47.2) | 0.658 |
| ≥1.035 | 30 | (58.8) | 21 | (41.2) |  |
| Total | 60 | (55.6) | 48 | (44.4) |  |
| ^a^Fisher’s exact test p-values were FDR-adjusted to account for multiple testing. | | | | | |
